# Sprouted Oats as Functional Ingredients: Biochemical Transformations, Nutritional Enhancement, and Health Implications

**DOI:** 10.3390/foods15111957

**Published:** 2026-06-01

**Authors:** Mary Inthavong, Regine Stockmann, Stefan Kasapis

**Affiliations:** 1 School of Science, Royal Melbourne Institute of Technology (RMIT), Bundoora West Campus, Plenty Road, Melbourne, VIC 3083, Australia; stefan.kasapis@rmit.edu.au; 2 Commonwealth Scientific and Industrial Research Organization (CSIRO), Agriculture and Food, 671 Sneydes Road, Private Bag 16, Werribee, VIC 3030, Australia; regine.stockmann@csiro.au

**Keywords:** oats, germination, sprouting, bioactive compounds, nutritional modifications

## Abstract

Over the past few decades, there has been a notable increase in the incorporation of sprouted oats into the human diet, driven by their perceived health benefits from enhanced nutritional qualities and bioactive contents. This review finds that sprouting consistently improves protein digestibility, phenolic content and GABA levels, while reducing anti-nutrients such as phytate—though the extent of enhancement is highly dependent on cultivar and germination conditions. These nutritional modifications contribute to potential health-promoting effects, including anti-inflammatory and chemopreventative properties, thereby opening opportunities in the food, cosmetic, pharmaceutical, and nutraceutical industries. However, a critical gap remains in limited human clinical trials validating these health benefits, and the influence of light spectra on the oat nutritional profile remains unexplored. A critical understanding of these germination-induced changes is essential for optimizing processing parameters and developing innovative products to meet evolving consumer demands for novel, health-focused food products.

## 1. Introduction

Driven by demands for nutrient-dense and sustainable foods, modern consumers are increasingly selective in their eating habits [1]. This trend is reflected in a shift away from animal-derived products, motivated by concerns over animal welfare, health, and sustainability [2,3,4,5,6,7]. Among cereal grains, oat (*Avena sativa* L.) stands out due to its widespread use in food applications from breakfast cereals to dairy alternatives [8] and its widely reported health benefits. Unlike other major cereals such as wheat or maize, oats possess a unique combination of β-glucan and avenanthramides (exclusive to oats), which are recognized to provide health benefits. These multifunctional benefits, which include cholesterol-reducing properties, anti-cancerous effects, and minimizing gastrointestinal problems, are attributed to its balanced nutritional profile and unique bioactive compounds [9,10,11,12].

To further enhance the nutritional value of oats, bioprocessing methods—including enzyme application, fermentation, and germination—have been explored. While enzymatic and fermentative processing can improve nutrient bioavailability and functionality, they involve added costs or specific food safety controls [13,14]. In contrast, controlled germination (commonly referred to as sprouting) offers a more streamlined approach. This process entails the physiological reactivation of dormant seeds under favorable conditions of water, oxygen, and temperature [15], applied for a specific duration to enhance the grain’s nutritional and functional properties for human consumption [16]. By utilizing the grain’s own endogenous enzymatic systems, sprouting represents a simpler, self-contained bioprocess.

The sprouting process, initiated by soaking under controlled humidity and temperature, leads to a cascade of biochemical changes. These include degradation of antinutrients, increases in free sugars, phenolic compounds, essential amino acids, soluble protein, and improvements in vitamin content [8,17,18,19,20]. However, if not correctly handled, packaged, and stored, sprouted grains are susceptible to microbial contamination and undesirable enzymatic degradation [21]. Previous reviews on sprouted grains have focused on individual nutritional components or general germination effects. A comprehensive synthesis of dose–response relationships between specific germination conditions (time, temperature, cultivar, light) is lacking. Quantitative dose–response data, human clinical evidence, and the effects of light spectra remain critically unexplored. This review aims to provide a comprehensive overview of the physicochemical and biochemical alterations in oats during sprouting, considering the impact of various processing conditions. Achieving this deeper understanding is essential for optimizing sprouting protocols and unlocking the potential of sprouted oats for the development of innovative functional foods and nutraceutical ingredients.

## 2. Literature Search Strategy

A comprehensive literature search was undertaken using Scopus, PubMed, Wiley, and ScienceDirect (1980–2026). To ensure broader coverage, Google Scholar was also consulted. The search strategy employed key terms and Boolean operators, including: (“sprouted oat*” OR “germinated oat*”) AND (“*Avena sativa*”) combined with (“nutrition*” OR “bioactive*” OR “phenolic*” OR “physicochemical” OR “functional property*”). Inclusion criteria were peer-reviewed articles, original research or reviews, published in English, reporting measurable outcomes of the sprouting process on oat composition or functionality. Exclusion criteria were conference abstracts, non-peer-reviewed sources, and studies not focused on germination. This is a narrative (traditional) review, not a systematic review. The reference lists of key articles were subsequently examined to identify additional relevant sources.

## 3. The Biochemical Phases of Germination

**Figure 1:**
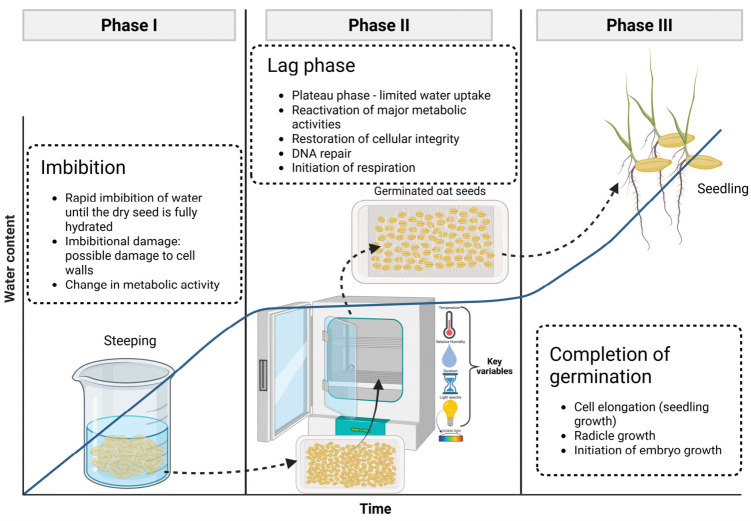
Schematic representation of the triphasic germination process. Note. The time course of water uptake typically characterizes the changes occurring during germination. In phase I, the seeds undergo imbibition, followed by Phase II, with minimal water intake, and increased metabolic activity. Phase III marks the establishment of seeds, accompanied by further water uptake, signifying the completion of germination. Created in BioRender. Inthavong, M. (2026) https://BioRender.com/9aqst8p, accessed on 20 May 2026.

Germination is a well-known bioprocessing technique that improves grains’ nutritional and functional properties through a triphasic physiological process (Figure 1): imbibition, a lag phase and radicle emergence. In oats, phase I (imbibition) involves the rapid uptake of water by the dry seed until its tissue achieves complete hydration, a process that can potentially cause imbibitional damage to the cell walls. This water uptake is critical for activating oat enzymes such as α-amylase and phytase. In Phase II, water intake plateaus as cellular integrity is restored, respiration initiates, and DNA repair occurs; this lag phase is characterized by peak metabolic activity. In oats, this phase sees the mobilization of storage compounds, including β-glucan and avenanthramide precursors. Phase III marks the completion of germination, with renewed water uptake driving cell elongation and radicle emergence. During this phase, storage nutrients are mobilized into simpler, usable compounds—such as sugars and amino acids—while cell division and DNA synthesis commence [22,23,24,25,26]. In sprouting oats, the embryo utilizes stored carbohydrates to facilitate growth, while hydrolytic enzymes break down complex substrates like fibers, starch, and proteins into simpler, more bioavailable forms. This enzymatic activity amplifies the content of free amino acids, sugars, bioactive compounds, vitamins, and antioxidant properties in the grain [8,19,27,28,29]. The extent of these biochemical changes is highly influenced by factors such as cultivar, germination conditions and laboratory methodologies [22].

## 4. Variables and Processing Conditions Influencing Germination Outcomes

### 4.1. Genotype (Cultivar)

The genetic makeup of the oat cultivar is a primary variable influencing the biochemical baseline from which sprouting begins and the trajectory of nutritional changes during germination. Different oat genotypes possess distinct initial concentrations and types of starches, proteins, β-glucans, and phenolic compounds which directly affect the substrate available for enzymatic activity during sprouting [30]. Consequently, the potential for nutrient enhancement such as the increase in free amino acids or antioxidant capacity is inherently linked to the chosen variety.

Furthermore, genotypic differences impact the physiological response to germination conditions. Studies indicate the germination rate, uniformity, and tolerance to abiotic stresses during the sprouting process (e.g., salinity in the soaking water) can vary significantly between cultivars [31]. Namely, some genotypes may exhibit delayed or reduced germination under suboptimal water potential, directly affecting the efficiency and yield of sprouting. Thus, selecting oat varieties not only for their agronomic traits but also for their germinability and sprouting responsiveness is crucial for optimizing the process. The interaction between genotype and sprouting environment highlights the need to tailor germination protocols to specific oat cultivars to achieve consistent and high-quality sprouted products.

Critically, genotype-by-environment (G × E) interactions must be considered when sourcing consistent raw material for sprouting. Howarth et al. [32] demonstrated that environmental conditions significantly altered the physical grain quality and composition (e.g., groat content, protein levels) of four winter oat varieties. This variability in the baseline composition of the grain directly influences the substrate available for enzymatic action during germination. Therefore, achieving a uniform, high-quality sprouted oat product requires not only selecting an appropriate genotype but also understanding and controlling the environmental factors that determine the grain’s initial biochemical profile.

### 4.2. Environmental Parameters

The controlled application of environmental parameters is fundamental in influencing the biochemical pathways activated during germination. Key factors include temperature, hydration, light, and abiotic stress, which do not act in isolation but form an interactive matrix that determines the rate of uniformity and the enhanced nutritional profile of sprouted oats.

#### 4.2.1. Temperature and Duration

Temperature is one of the most important variables controlling the rate and direction of metabolic activity during oat sprouting, with optimal ranges being highly specific to the targeted nutritional outcome. For the enhancement of antioxidant capacity and soluble protein, a moderate temperature range of 16 to 20 °C is most effective. This is demonstrated by Tang et al. [33], who reported increases of 13.52 mg g^−1^ total phenolics and 28.19 mg g^−1^ soluble protein after steeping at 18 °C and germinating at 20 °C for 48 h, and by Aparicio-García et al. [34] who identified cultivar-specific optima of 16 °C for 216 h for ‘Barra’ oats and 18 °C for 156 h for ‘Meeri’ oats. In contrast, to rapidly activate hydrolytic metabolism and increase sugars, a slightly warmer range of 20 to 25 °C is preferable, as shown in the ‘Gehl’ cultivar, where it boosted ascorbic acid, reducing sugars and amylase activity within a three-day process [19]. Meanwhile, the effective degradation of antinutrients like phytate varies amongst cultivars, ranging from a single incubation at 15 °C [35] to a complex multi-stage malting process involving 5 days at 11 °C followed by incubation at 37 to 40 °C to achieve an 80 to 85% reduction [36]. Consequently, the time-temperature must be purposefully selected to steer the sprouting process towards the desired biochemical pathway and nutritional profile.

#### 4.2.2. Hydration

Water acts as both the initial trigger and the essential medium for all biochemical mobilization during sprouting. The two critical hydration phases are: initial imbibition for metabolic reactivation (Phase I) and sustained uptake to support radicle growth and reserve mobilization (Phase III) [23]. The efficacy of this hydration is determined by seed permeability and the composition of the soaking medium. Simply providing adequate moisture is not sufficient; maintaining optimal hydration throughout the germination period is crucial to support the continuous enzymatic activity responsible for improving the physicochemical and nutritional properties of the oat [27]. Insufficient or excessive water can lead to uneven germination, microbial spoilage, or suboptimal nutrient enhancement.

#### 4.2.3. Light Exposure

The presence of light is a critical regulator of plant development, influencing physiological and metabolic pathways that determine growth and nutritional composition. Exposure to light initiates de-etiolation, a developmental transition that prepares seedlings for efficient photosynthetic activity and influences the shift from vegetative to reproductive development [37]. Beyond morphological changes, light quality and intensity have been shown to modulate the accumulation of specific bioactive compounds, allowing targeted enhancement of nutritional attributes through spectral manipulation [22].

Despite the growing interest in sprouted oats, the influence of light and dark germination conditions remains largely unexplored in this matrix. Most of the available evidence comes from studies on other sprouted seeds and grains, not oats—a limitation that should be acknowledged. Nevertheless, insights can be drawn from studies on other sprouted seeds and grains. Mastropasqua et al. [38] demonstrated that darkness resulted in higher dry matter retention in radish, soybean, mung bean and pumpkin seeds, likely reflecting reduced utilization of storage reserves within the cotyledons. Contrarily, exposure to white, red, and blue light promoted mobilization of these reserves, leading to decreased dry matter but enhanced synthesis of antioxidant compounds, including vitamin C, carotenoids, chlorophylls, and anthocyanins. In agreement with this metabolic shift, total sugar content, particularly sucrose, was significantly higher in dark-germinated mung beans and soybeans.

The importance of utilizing the light spectrum during germination has been further supported by Puccinelli et al. [39], who reported that blue light exposure significantly enhances the nutritional quality of flaxseed sprouts and microgreens by increasing flavonoids, total phenolics, chlorogenic acid, and overall antioxidant capacity. Interestingly, Van Hung et al. [40] observed greater dry matter loss under dark germination in mung beans due to increased metabolic and enzymatic activity. The higher diastatic, α-amylase and protease activities accelerated the breakdown of stored reserves to support respiration and sprout growth in the absence of photosynthesis. As a result, dark-germinated seeds showed greater increases in phenolic content, flavonoids and antioxidant capacity and enzyme inhibitory activities when expressed on a dry weight basis. Contrastingly, light-exposed sprouts exhibited elevated levels of essential amino acids, including lysine, histidine and methionine, as well as high proportions of unsaturated fatty acids in which they highlight a balance between reserve mobilization and nutritional composition. Consistent trends have been observed in cereal sprouts, where Xiang et al. [41] noted that light-germinated sweet corn sprouts accumulated significantly higher levels of phenolic compounds, flavonoids and antioxidant activity compared to those grown in the darkness, with comparisons made on a dry matter basis. Additionally, Samuolienė et al. [42] observed that light-emitting diode (LED) illumination enhanced the antioxidant properties of sprouted seeds relative to dark conditions, with green (510 nm) and amber (595 nm) light specifically increasing α-tocopherol and vitamin C concentrations in lentil, wheat and radish sprouts, suggesting a direct light modulation of antioxidant metabolism.

While these findings cannot be directly extrapolated to oats without experimental validation, they provide a strong rationale for investigating light conditions in oat sprouting. The similar cereal background of wheat and corn, and the consistent trends across diverse cultivars, suggest that light is likely to influence oat bioactive profile, though the magnitude and direction of effects may be cultivar and condition dependent. Collectively, these findings emphasize that both light exposure and spectral composition play a pivotal role in shaping the nutritional bioactive profiles of sprouted seeds. However, the effect on dry matter is not uniform: while some studies report higher dry matter retention in darkness [38], others, such as Van Hung et al. [40] observed greater dry matter loss under dark conditions due to increased metabolic activity. Dark germination generally favors sugar accumulation, whereas light, particularly specific wavelengths, appear to promote the biosynthesis of antioxidant and health-promoting compounds. These insights stress the importance of considering light conditions when optimizing germination strategies for sprouted grains, including oats, to maximize their nutritional and functional potential. Direct studies on oats are now needed to confirm these trends and establish cultivar-specific light protocols.

#### 4.2.4. Other Abiotic Stresses

As discussed above, germination is profoundly influenced by environmental parameters such as temperature, water availability, and light. In addition to these factors, abiotic stresses including salinity and relative humidity can substantially influence both the seedling development and the accumulation of bioactive compounds during sprouting [22].

Salinity represents one of the most critical abiotic constraints during early seedling development, a stage characterized by heightened sensitivity to osmotic and ionic stress [22]. Although its effects have been extensively studied in crop establishment, the influence of salinity on the phytochemical composition of oat and cereal sprouts remains poorly understood. Evidence from related species suggests that salt stress exerts a dual effect, whereby excessive salinity impairs germination, while moderate levels can stimulate secondary metabolite synthesis. Xiong et al. [43] demonstrated that elevated salinity levels delayed barley germination by reducing starch mobilization efficiency and embryo growth, primarily through the inhibition of α-amylase activity by downregulation of *Amy1*, *Amy2*, and *Amy3* genes. In contrast, low salinity (1 to 20 mM sodium chloride, NaCl) has been reported to stimulate the synthesis of phenolic components. Trasmundi et al. [44] observed increased phenolic content in durum wheat seedlings germinated under 200 mM NaCl, whereas higher concentrations (250 to 300 mM) negatively affected germination performance. Similar trends were reported by Falcinelli et al. [45], who identified NaCl concentrations as optimal for maximizing polyphenol content and antioxidant capacity in sprouted wheatgrass, einkorn and emmer. Comparable stress-induced enhancements have been reported in non-cereal species, such as *Nepeta racemosa* Lam., where salinity-driven increases in phenolic compounds and flavonoids were strongly correlated with elevated antioxidant capacity [46]. Collectively, these findings point to the existence of an optimal salinity threshold that promotes phytochemical synthesis without compromising germination.

In addition to salinity, relative humidity plays a pivotal role in regulating germination performance and metabolic activity. Figueroa-Pérez et al. [47] stated that oats germinated between 60 and 65% relative humidity achieved high germination rates (97 to 100%) and improved nutrient composition. Notably, the combination of 30 °C and 65% relative humidity resulted in the highest accumulation of phenolic acids, avenanthramides and lignans, highlighting the synergistic influence of temperature and humidity on phytochemical profiles in sprouted oats. However, prolonged exposure to high humidity can increase the risk of fungal contamination, including *Fusarium poae*, which poses significant food and safety concerns [48]. Indeed, while high humidity (85 to 99%) is widely used to promote rapid metabolic reactivation and bioactive accumulation [20,49,50,51,52,53], it must be carefully managed against the risk of microbial spoilage. This process is accompanied by hormonal changes such as abscisic acid and gibberellic acid levels [21].

These interactive effects of relative humidity and light have been demonstrated in legume sprouts. Amitrano et al. [54] investigated that variations in relative humidity primarily influenced the physical development of mung bean sprouts, whereas light exposure modulated antioxidant compound accumulation. Specifically, the combination of light (150 μmol photons) and low relative humidity (60%) enhanced antioxidant production, while sprouts grown in darkness exhibited reduced size but the highest overall antioxidant capacity. These observations are consistent with oat-based findings reported by Figueroa-Pérez et al. [47] and reinforce the notion that multiple abiotic factors interact to shape both growth and nutritional outcomes during germination.

## 5. Changes in Chemical Composition During Germination

Sprouting induces profound biochemical transformations across all major nutritional fractions of the oat grain. These changes are driven by the activation of endogenous enzymes during germination, with the specific enzyme system and substrate varying by compound class—including α-amylase for starch, β-glucanase for β-glucan, proteases for protein, phytase for phytate, lipase for lipids, glutamate decarboxylase (GAD) for GABA, and phenylalanine ammonia-lyase (PAL) for phenolics. While the enzyme systems differ, the underlying principle of enzyme-mediated nutritional transformation is consistent (Section 5.1, Section 5.2, Section 5.3, Section 5.4, Section 5.5 and Section 5.6). A comprehensive summary of the effect of germination on the nutritional properties in oats is provided in Table 1.

Germination-induced changes in bioactive compound content arise through two complementary mechanisms: the mobilization of existing stored forms via enzymatic release, and de novo biosynthesis of new compounds in the developing seedling. Both mechanisms operate simultaneously across the compound classes discussed below, though their relative contributions vary by compound and germination conditions.

Throughout Section 5.1, Section 5.2, Section 5.3, Section 5.4, Section 5.5 and Section 5.6, a consistent theme emerges: the magnitude of germination-induced changes is highly dependent on cultivar genotype and the specific time-temperature protocol employed. This cultivar and condition dependency applies to all nutritional outcomes discussed and will not be restated in full for each subsection. Changes in specific nutritional composition are summarized in Table 2.

Finally, across all compound classes discussed in Section 5.1, Section 5.2, Section 5.3, Section 5.4, Section 5.5 and Section 5.6, a critical and consistent gap remains: human intervention trials directly measuring the bioavailability and functional efficacy of germination-enhanced compounds are lacking. Existing evidence is predominantly derived from in vitro models and animal studies, which limits direct translation to human nutritional recommendations. The functional efficacy and clinical validation of germination-enhanced compounds are addressed in Section 7.

### 5.1. Impact of Sprouting on Carbohydrate Composition

Sprouting fundamentally alters the carbohydrate profile of oats, primarily through the enzymatic breakdown of complex storage polysaccharides into simpler, more bioavailable sugars [57]. Starch levels typically range from 51 to 65% of the oat kernel and are primarily stored in the endosperm [11,17] as semi-crystalline starch granules [69].

The activation of endogenous α-amylase is the principal catalyst, hydrolyzing these granules and leading to a general decrease in total starch content. This enzymatic activity shows a clear temporal and thermal dependence. For example, germination at 16 °C for 6 days had an average decrease in starch by 4.5% in hull-less oats, a change accompanied by increased α-amylase activity [55]. Tian et al. [56] observed a similar correlation in ‘Shaanxi’ oats, with a pronounced 20 to 60% reduction in starch and a rise in α-amylase activity after 48 h. This breakdown is reflected at a molecular level in a corresponding reduction in the amylose content, as observed by Li et al. [29], who reported a 17% decrease in ‘Choyang’ oats at 25 °C for 60 h. In contrast, Jiménez-Pulido et al. [50] noted a slight starch increase alongside a decrease in dietary fiber at 21 °C for 5 days. This anomaly highlights the complexity of co-occurring cell wall degradation and the influence of analytical methods on reported outcomes.

Despite such anomalies, the trend across studies is clear: starch hydrolysis drives a net increase in soluble sugars. Lineback and Ponpipom [57] documented a six-fold increase in total free sugars at 15 °C for 14 days, accompanied by a 61-fold increase in α-amylase activity, while Ding et al. [20] observed substantial rises in fructose, glucose, maltose, and sucrose at 24 °C within 48 to 72 h. The profile of individual sugars can vary; for instance, while glucose and maltose consistently rise, fructose levels may decline and recover in some cultivars or conditions, suggesting differential metabolic fates for the initial hydrolysis products [61]. The efficiency of this conversion is temperature-dependent, with optimal α-amylase activity and sugar production reported in the 20 to 25 °C range. The increased degradation of starch in oat grains involves the de novo synthesis of α-amylase [19]. This shift from complex starch to simple sugars and modified fibers has direct implications for the nutritional functionality, glycemic response, and sensory properties of sprouted oat products.

#### 5.1.1. Dietary Fiber

Oats are a rich source of dietary fiber; a non-digestible carbohydrate associated with reduced risk of cardiovascular and gastrointestinal diseases [70,71,72]. Sprouting induces significant changes in both the total content and the solubility profile of fiber.

##### Total Fiber Content and Insoluble Dietary Fiber

The overall dietary fiber content of oats often increases during germination, primarily due to the respiration and loss of other dry matter components like starch [35]. However, the most nutritionally relevant change is the shift in the ratio of insoluble dietary fiber (IDF) to soluble dietary fiber (SDF). IDF, which comprises cellulose, lignin, and resistant starches, shows a variable response to sprouting. For instance, Hübner et al. [35] reported a rise in IDF to approximately 30% after prolonged germination (144 h), Huang et al. [67] noted a significant decrease by 53% at 23 °C for 216 h for common oats, while Aparicio-García et al. [18] found no statistical change at 18 °C for 96 h for ‘Meeri’ oats, consistent with cultivar and condition dependency established above. This variability can be attributed to enzymatic hydrolysis of cell wall components like cellulose and hemicellulose to produce soluble sugars for sprout growth during the germination process [67]. In contrast, the more consistent and consequential change occurs in the soluble dietary fiber (SDF) fraction, particularly β-glucan. This viscous, fermentable fraction is pivotal for metabolic health benefits such as cholesterol reduction and prebiotic activity [70,71].

##### β-Glucan

β-glucan is the most valued SDF in oats, comprising 3 to 8% of its dry weight and responsible for many of its functional and health-promoting properties [73,74,75,76,77]. In non-sprouted oats, high molecular weight β-glucan forms viscous solutions in the small intestine, which is associated with cholesterol lowering, glycemic control, and immune modulation [75].

The literature clearly reveals that sprouting consistently reduces β-glucan content via activation of endogenous β-glucanase enzymes. The extent of this degradation is a direct function of time and temperature. The temporal effect is demonstrated by an inverse relationship between germination and β-glucan content. Near-complete degradation has been observed after extended periods of 6 to 9 days [35,55,58,67], while significant reductions of 47 to 64% are reported at durations of 156 to 216 h [34], and approximately 44% with 40 to 50 h [66]. Simultaneously, the thermal effect modulates the rate of this hydrolysis. Krapf et al. [19] and Aparicio-García et al. [34] found that temperatures between 18 and 20 °C resulted in the most significant degradation of β-glucan, with markedly less loss at cooler temperatures (10 to 14 °C), confirming that β-glucanase activity follows typical enzymatic kinetics where warmer conditions accelerate the reaction.

Critically, sprouting reduces not only the quantity but also its molecular weight (M_w_). A key determinant of its viscosity and health-promoting efficacy, this depolymerization has significant functional consequences [78,79,80,81,82]. While studies on M_w_ changes during germination are limited, available evidence confirms this depolymerization. Wilhelmson et al. [58] observed a decrease in M_w_ from 2.4 × 10^6^ to 1.5 × 10^6^ in ‘Lisbeth’ oats over six days at 15 °C, with similar trends in ‘Veli’. This pattern of high-M_w_ breakdown is consistent with the action of endo-β-glucanases. Research in similar bioprocesses, such as oat sourdough fermentation with *Lactobacillus plantarum*, demonstrates a shift from high M_w_ polymers (>10^5^ g/mol) to a dominance of low-M_w_ fragments (<10^5^ g/mol) as hydrolysis proceeds [83]. Sprouting therefore compromises β-glucan both quantitatively and qualitatively, and both dimensions must be considered when evaluating the functional quality of sprouted oats.

##### Synthesis and Implications for Processing Fiber

The transformation of oat fiber during sprouting presents a compromise. While the process may increase total fiber and potentially enhance other bioactive compounds, it unavoidably depletes β-glucan, a key functional ingredient. This creates a critical optimization challenge for food technologists. The choice of germination parameters (time and temperature) must be strategically aligned with the product’s nutritional goals: maximizing β-glucan preservation requires shorter, cooler sprouting protocols, whereas pursuing other benefits may justify its partial degradation. Subsequent studies should prioritize precisely mapping this compromise to enable the tailored design of sprouted oat ingredients.

### 5.2. Impact of Sprouting on Protein and Amino Acid Composition

Germination fundamentally transforms oat protein through the activation of endogenous proteases. These enzymes hydrolyze storage proteins (globulins and prolamins) into soluble, digestible albumins, breaking down complex storage proteins to provide simpler peptides and free amino acids for the growing embryo [50,59,60,84]. Furthermore, as the seed mobilizes carbohydrate reserves to fuel growth, respiratory dry matter loss causes a relative concentration of existing nitrogen within the grain. The seed functions as a closed system for nitrogen. Consequently, the calculated protein content (nitrogen × 6.25) increases per unit mass, even though total nitrogen remains relatively constant [85].

#### 5.2.1. Protein

While oats contain a relatively high protein content (15 to 20%) compared to other cereals [11,86,87], reported changes in total protein content during sprouting are variable. Some studies report net increases in protein content from ~19 to 22% [56], or by 11 to 15% [18,34]. As noted in Section 5.2, this apparent increase reflects nitrogen concentration from respiratory dry matter loss rather than de novo protein synthesis [85]. This variability is consistent with the cultivar and condition dependency established above. Some studies note slight decreases in ‘Chimene’ oats [50], or significant reductions up to 50% in common oats [67]. This variability can be attributed to the balance between the initial concentration effect and the eventual utilization of amino acids for seedling growth, which ultimately causes a net decline in protein content during prolonged sprouting [88].

Beyond changes in total content, germination enhances the functional quality of oat protein through improved digestibility [14]. While this effect is well-documented in other cereals and legumes [89,90,91,92], recent evidence confirms it in oats. Bagarinao et al. [93] demonstrated that germinating *Avena sativa* var. ‘L5’ at 25 °C enhanced gastric-phase protein digestibility by 5.8-fold, alongside increases in free α-amino groups and water extractable proteins, and reductions in antinutritional compounds such as phytic acid and trypsin inhibitor activity. Consequently, through the combined effects of proteolysis, reduced antinutritional factors, and improved solubility. This suggests that germination enhances both the digestibility and nutritional quality of oat protein compared to its ungerminated counterpart.

#### 5.2.2. Amino Acids

Germination-induced hydrolysis of proteins influences both the free and total amino acid profile, particularly increasing the concentration of limiting essential amino acids. The essential amino acids required by humans are histidine, isoleucine, leucine, lysine, methionine, phenylalanine, threonine, tryptophan, and valine. In both raw and sprouted oats, lysine remains the limiting amino acid [94]. The most consistent improvements are observed in lysine, threonine, and histidine across diverse cultivars. In common milled oats, lysine content increased from 4.4 to 5.3 g/16 g N after germination [59], and free amino acids can increase nearly 10-fold [56]. Studies on specific cultivars reinforce this: significant increases in most free amino acids were reported in ‘Baiyan II’ and ‘Bayou I’ oats [62], while ‘Meeri’ oats showed improvements in methionine, cystine, and phenylalanine in sprouted flour [18]. Notably, histidine exhibited a statistically significant 20% increase [60], a finding confirmed by Tang et al. [33]. While the magnitude of increase varies by cultivar, consistent with the pattern established above, sprouting enhances the availability of essential amino acids, with lysine remaining as the limiting amino acid.

##### Synthesis and Implications for Protein Quality

Oats are recognized as a high-quality protein source among other major cereals [60,86,95]. Sprouting transforms oat protein from a stored, complex form into a more simplified and bioavailable nutritional source. The process may not guarantee a higher protein percentage due to competing metabolic pathways and cultivar-specific responses, but it reliably enhances protein quality by boosting levels of critical essential amino acids and increasing solubility and digestibility. Longer germination durations (120 to 216 h) and moderate temperatures (16 to 20 °C) favor greater accumulation of essential amino acids. While sprouting improves oat protein quality, clinical gaps remain regarding the cultivar-specific protein digestibility responses and to validate these improvements through human clinical trials assessing post-meal amino acid bioavailability and satiety responses.

#### 5.2.3. γ-Aminobutyric Acid

**Figure 2:**
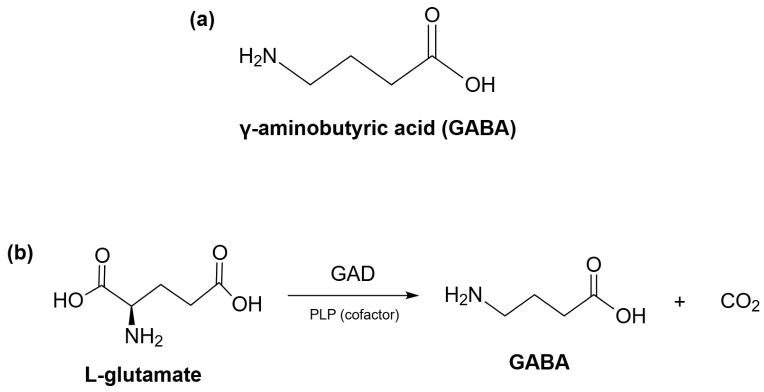
Chemical structures of (**a**) γ-aminobutyric acid (GABA) and (**b**) its biosynthesis from L-glutamate via glutamate decarboxylase (GAD) with pyridoxal phosphate (PLP) as cofactor, producing CO_2_ as a by-product.

γ-aminobutyric acid (GABA) is a non-protein amino acid that functions as a major inhibitory neurotransmitter in mammals and as a stress-response metabolite in plants (Figure 2). In humans, dietary GABA is associated with beneficial effects such as lowered blood pressure, inhibiting cancer cell proliferation, anti-anxiety activity, and cognitive enhancements [62,96,97]. It is synthesized in plants primarily via the α-decarboxylation reaction of L-glutamic acid or its salts, catalyzed by glutamate decarboxylase enzyme (GAD) [98,99,100].

Oat germination is a highly effective bioprocess for enriching GABA. Research consistently shows a multi-fold increase in GABA content during germination. Ding et al. [20] reported an exponential increase with levels reaching up to 31 times the concentration in raw oats (9.47 to 297.69 µg 100 mg^−1^) after 96 h at 24 °C. Similarly, Xu et al. [62] observed an increase from 1.41 to 19.62 mg g^−1^ in ‘Bayou I’ over 72 h at 20 °C, and Aparicio-García et al. [18] documented a 3.2-fold increase (16.71 to 54.92 mg 100 g^−1^) in ‘Meeri’ oats after 96 h at 18 °C. The magnitude of this increase is influenced by germination temperature, duration, and cultivar. Longer germination periods and higher temperatures promote greater GABA accumulation, likely due to sustained GAD activity. This is driven by metabolic shifts in the GABA shunt pathway, including increased availability of precursor organic acids such as succinate and α-ketoglutarate [20,62]. This variation is consistent with the cultivar and condition dependency established above. Therefore, to maximize the GABA content of sprouted oats for development of functional foods, process optimization must target extended germination times and select for high-response cultivars.

##### Synthesis and Implications for GABA

Sprouting significantly enhances the concentration of GABA in oats, with optimal accumulation occurring at germination durations of 72 to 96 h and temperatures of 18 to 24 °C. Beyond the biochemical enrichment, emerging evidence supports the functional efficacy of GABA for human health. Xu et al. [101] demonstrated that oral GABA supplementation (20 mg kg^−1^) in chronically stressed mice significantly alleviated anxiety-like behaviors while restoring GABA levels in the prefrontal cortex.

The oral bioavailability of GABA in humans has been demonstrated at the systemic level; however, its ability to cross the blood–brain barrier (BBB) remains limited and is subject to ongoing debate. Current evidence suggests that, although small amounts of GABA may cross the BBB in animal models, there is insufficient human data to confirm meaningful central nervous system uptake [102,103]. Thus, while sprouted oats are a rich dietary source of GABA, enhanced GABA does not equate to bioactive delivery in the human central nervous system. The potential of sprouted oats as a viable natural source of bioactive GABA for functional food applications requires validation through clinical trials that directly measure GABA absorption, BBB penetration, and neurological outcomes, to confirm these neuroimmune mechanisms using sprouting oat products with well-characterized GABA content.

### 5.3. Impact of Sprouting on Lipids

Oats are a notable lipid source among cereals, with a content of 3 to 18% comprising primarily palmitic, oleic, and linoleic acids [104]. Lipids in the form of triacylglycerol (TAG) are generally stored in the plant seed until germination, where these reserves are hydrolyzed by lipases to produce free fatty acids and glycerol [105]. Sprouting induces a transformation in the fatty acid profile, steering towards a more polyunsaturated composition. The direction of change is consistent, though the magnitude is influenced by germination conditions and cultivar.

Research under various sprouting conditions confirms a trend of increasing polyunsaturated fatty acids (PUFA), consistent with the cultivar-dependent pattern established above. In ‘Meeri’ oats sprouted at 18 °C for 96 h, Aparicio-García et al. [18] reported significant increases in palmitoleic, linoleic, and α-linolenic acids, resulting in a higher total PUFA and lower monounsaturated fatty acid (MUFA) levels. Similarly, in a study of common oat germinated at 20 °C for 72 h, Al-Taher and Nemzer [63] observed a 2.2-fold increase in total lipid content, with significant increases in linolenic acid and lignoceric acid, contributing to an improved omega-6/omega-3 ratio. In contrast, a study on the ‘Chimene’ cultivar at 21 °C for 120 h recorded a different profile: increases in MUFA and SFA (20%) driven by a rise in oleic acid, alongside a slight decrease in linoleic acid [50]. The predominant biochemical driver for a PUFA shift is the activation of desaturase enzymes during germination, which convert oleic acid (a MUFA) into linoleic and linolenic acids (PUFA) [106]. This mechanism explains the frequent observation of a decrease in oleic acid, as noted by Aparicio-García et al. [18], simultaneously with PUFA gains. The divergent result in ‘Chimene’ likely reflects genotypic differences in lipid metabolism, consistent with cultivar dependency established above [107].

#### Synthesis and Implications for Lipids

Sprouting induces significant changes to the oat lipid profile, generally shifting the composition towards more PUFAs through the activation of desaturase enzymes [106]. While this enhances nutritional quality by increasing beneficial unsaturated fatty acids, the elevated lipid content, and PUFAs introduce a practical trade-off: increased susceptibility to oxidation, which may produce off-flavors and reduce consumer acceptance of sprouted oat products [18]. To optimize the lipid profile of sprouted oats, processors must balance nutritional enhancement against oxidative stability. A key priority for future work is establishing cultivar-specific lipid responses and developing integrated post-sprouting strategies (e.g., modified atmosphere packaging) to preserve the enhanced fatty acid profile while maintaining sensory quality.

### 5.4. Impact of Sprouting on Phytate

Phytate (myo-inositol phosphate) is the primary phosphorus storage compound in oat grains and a potent antinutrient that binds minerals like iron and zinc, reducing their absorption in the human gut [108,109]. Sprouting activates endogenous phytase enzymes, which hydrolyze phytate into lower inositol phosphates with weaker mineral-chelating capacity, thus representing a natural strategy to improve mineral bioavailability [109].

The effectiveness of phytate degradation is highly dependent on germination conditions, particularly time and temperature. Under standard sprouting protocols, significant decreases are common. Hübner et al. [35] highlighted a reduction from 0.683% to 0.467%, and Tian et al. [56] observed a more pronounced drop from 0.35% to 0.11%. These reductions are driven by increased phytase activity, which mobilizes phosphorus for the growing seedling. However, optimized, prolonged protocols designed to maximize phytase activity can achieve near complete phytate elimination. The foundational work by Larsson and Sandberg [36] demonstrates this potential, achieving an 80 to 85% reduction through a specialized germination protocol: a prolonged germination phase of 5 to 7 days at 11 °C, followed by a soaking step at 37 °C and pH 5. This protocol is designed to maximize phytase synthesis and activity. Conversely, shorter or less intensive sprouting yields more modest results such as the 12% reduction (0.94 to 0.83 g 100 g^−1^) presented in Jiménez-Pulido et al. [50].

#### Synthesis and Implications for Phytate

Sprouting consistently initiates phytate degradation through the activation of endogenous phytase enzymes. However, the extent of this reduction varies considerably with germination conditions and cultivar, with studies demonstrating a spectrum of outcomes: standard protocols achieved moderate reductions (12 to 45%), while optimized and prolonged protocols achieved 80 to 85% reduction. To reliably enhance the mineral bioavailability of sprouted oat products, process optimization must favor longer germination durations and tailored time-temperature protocols that sustain high phytase activity. Conversely, processors must balance phytate reduction against other nutritional objectives, as extended sprouting may degrade desirable components such as β-glucan. Subsequent studies should prioritize developing integrated germination protocols that optimize phytate reduction while preserving other bioactive compounds, allowing for the tailored design of sprouted oat ingredients with maximized nutritional value.

### 5.5. Impact of Sprouting on Minerals

Oats contain a well-balanced profile of essential minerals, including potassium, calcium, magnesium, iron, and zinc [11,110]. While sprouting does not synthesize new minerals, it can alter their measured concentrations, and more importantly, their nutritional availability. As established in Section 5.4, phytase activation during germination hydrolyses phytate, releasing bound minerals and improving their bioavailability [14].

Documented changes in total mineral content are variable and often minor, reflecting the closed system of the seed. Hübner et al. [35] observed slight changes in most minerals under different germination conditions, with the exception of variable copper levels. For example, calcium increased from 20.8 to 32.4 mg 100 g^−1^ (12 °C, 96 h), magnesium from 114 to 130 mg 100 g^−1^ (15 °C, 96 h), and copper from 0.2 to 1.5 mg 100 g^−1^ (15 °C, 96 h).

Similarly, Aparicio-García et al. [18] noted increases in sprouted ‘Meeri’ oats: calcium (51.3 to 68.3 mg 100 g^−1^), magnesium (158.7 to 191.3 mg 100 g^−1^), sodium (0.6 to 14.4 mg 100 g^−1^), and small increases in iron and zinc, alongside decreases in potassium and manganese. This variability likely stems from differences in cultivar, analytical method, and the loss of dry matter (primarily carbohydrates), which can relatively concentrate mineral fractions [111].

#### Synthesis and Implications for Minerals

The most significant impact of sprouting on mineral nutrition is indirect. As established in Section 5.4, sprouting activates phytase enzymes that degrade phytate, the primary mineral-chelating antinutrient in oats. Therefore, even if total mineral content remains stable, sprouting fundamentally improves the bioavailability of key minerals like iron and zinc by liberating them from insoluble phytate complexes. Thus, the nutritional value of sprouted oats regarding minerals is best understood through two factors: (1) possible modest fluctuations in total content due to metabolic shifts and dry matter loss, and (2) an improvement in bioavailability due to phytate reduction, the latter being the more impactful for nutritional planning. Critical gaps remain, particularly regarding quantifying the bioaccessibility of key minerals using in vitro digestion models across varied germination conditions and cultivars. Human intervention trials assessing mineral absorption from sprouted oat products are also needed to validate these improvements.

### 5.6. Impact of Sprouting on Phenolic Compounds and Antioxidant Activity

#### 5.6.1. Phenolic Compounds

Phenolic compounds, such as ferulic and *p*-coumaric acids in oats, are potent antioxidants linked to a reduced risk of chronic diseases [112,113]. The most abundant bound phenolic acids in oat products are ferulic, caffeic, and sinapic acid [114]. Sprouting induces a profound and consistent transformation in their profile, significantly enhancing both their total content and crucially their bioavailability.

Unambiguously, studies have highlighted a major increase in total phenolic content during germination, often by 2.5 to over 4-fold. As shown, in ‘Meeri’ oats sprouted at 18 °C for 96 h, total soluble phenolics increased from 202.92 to 507.39 mg GAE 100 g^−1^ [18]. Likewise, Tian et al. [56] observed more than 4-fold increase, from 0.19 to 0.90% DW, after 120 h at 16 °C. Recent studies confirm this trend across different materials: in common oats, total phenolics peaked at 4.22 ± 0.20 mg GAE g^−1^ after 9 days at 23 °C [67], while in the germ/shoot fraction of ‘Kamil’ and ‘Noni’ oats free phenolic compound increased 2.6-fold (3.90 to 10.15 mg FAE g^−1^ DW) and 5.2-fold (2.31 to 12.04 mg FAE g^−1^ DW), respectively, reaching maximum values at 120 h and 20 °C [52]. The authors have noted that the shoot tissue became a significant source of bound phenolics during germination due to its increasing dry weight, whereas increases in the kernel were more modest.

The underlying biochemical shift is even more significant: the redistribution from bound to free phenolic forms. Xu et al. [49] quantified this shift in naked oats, showing that while bound phenolics decreased from 1.23 to 0.87 mg GAE g^−1^ DW during germination at 16 to 20 °C, free phenolics surged from 0.43 to 1.74 mg GAE g^−1^ DW. This occurs because germination activates cell wall-degrading enzymes that liberate bound phenolics into free, bioaccessible forms [18,49]. De novo synthesis may further augment this release. The redistribution is nutritionally significant because free phenolics are more readily absorbed in the gastrointestinal tract than their bound counterparts [115]. The non-significant rise noted by Kruma et al. [64] after only 48 h confirms that this transformation is both time- and cultivar-dependent, consistent with the pattern established above.

The profile of individual phenolic acids also shifts during germination, with implications for antioxidant capacity. Xu et al. [49] also examined four common phenolic acids in ‘Baiyan II’ oats (gallic, ferulic, *p*-coumaric, and caffeic acid). Gallic and caffeic acid peaked at 24 h before declining, while *p*-coumaric and ferulic acid increased by 255% and 186%, respectively, by 48 h. Similarly, Živković et al. [52] observed a significant increase in bound trans- and cis-ferulic acid in shoots in both common and naked oats, alongside a dramatic decline of trans-*p*-coumaric acid. While the increase in bound phenolics may appear to contradict the broader trend of phenolic mobilization, this finding reflects tissue-specific differences: the shoot tissue actively synthesizes and deposits new bound phenolics during growth, whereas the kernel primarily releases existing bound forms into soluble fractions [52]. This highlights that the balance between bound and free phenolics is tissue-dependent and influenced by metabolic priorities of the developing seed.

#### 5.6.2. Antioxidant Activity

The increase in soluble phenolic compounds during sprouting directly translates into a significant enhancement of antioxidant activity. This enhancement reflects the mobilization of free phenolics described in Section 5.6.1, as these liberated forms possess radical-scavenging and metal-chelating capacity via their hydroxyl groups [18].

Consequently, quantitative assays consistently report a multi-fold increase in antioxidant capacity following germination, linking to the phenolic shifts detailed in Section 5.6.1. Xu et al. [49] demonstrated that in ‘Baiyan II’ oats, despite the decline in the antioxidant capacity of bound phenolics, the activity of free phenolics surged 5.4-fold, driving a 1.8-fold increase in total antioxidant capacity. Similarly, in common oats, Huang et al. [67] observed a dramatic 10-fold and 6-fold increase in DPPH and ABTS radical scavenging capacity, respectively at 23 °C for 216 h. Cultivar-specific studies confirm this trend: in ‘Meeri’ oats, Aparicio-García et al. [18] measured a 3-fold increase (585.26 to 1744.27 mg TE 100 g^−1^), and in ‘Noni’ oats shoots, Živković et al. [52] reported a 2.8-fold, peaking at 72 h at 20 °C. This enhancement is optimized by specific germination conditions; Aparicio-García et al. [34] identified that temperatures of 16 to 18 °C combined with extended durations (156 to 216 h) yielded the highest activity, as this window allows for substantial phenolic mobilization without progressing to advanced stages where these compounds may themselves be metabolized. This finding is supported by other studies that noted significant increases under various sprouting conditions [33,64,66].

##### Synthesis and Implications for Phenolics and Antioxidant Activity

Sprouting transforms oat phenolics through the release of existing bound phenolics from the kernel via cell wall degradation, and the de novo synthesis of new phenolics in growing shoot tissues [52]. The net effect is a significant increase in total phenolic content and a shift towards more bioavailable free forms, which directly translates into enhanced antioxidant capacity. This transformation fundamentally improves the nutritional quality of oats. By converting insoluble, bound antioxidants into soluble, bioactive free forms, sprouting not only increases the quality of these compounds but also activates their functional capacity to combat oxidative stress. To optimize this beneficial shift, germination protocols should generally exceed 48 h under favorable temperatures to allow for complete enzymatic interactions and phenolic mobilization.

Collectively, sprouting naturally amplifies the antioxidant potential of oats. A key priority for future work is establishing cultivar-specific phenolic responses to predict optimal germination conditions, quantifying bioaccessiblity of free phenolics using in vitro digestion models, and validating the functional efficacy of sprouted oat phenolics in human intervention trials targeting oxidative stress markers.

#### 5.6.3. Avenanthramides

Avenanthramides are unique, bioactive phenolic compounds found almost exclusively in oats, renowned for potent anti-inflammatory and antioxidant properties linked to cardiovascular health and colon cancer [114]. In non-sprouted oats, avenanthramides are present in relatively low concentrations ranging from 2 to 82 µg g^−1^ [116]. Sprouting is one of the most effective processes for enriching these valuable compounds [65].

**Figure 3:**
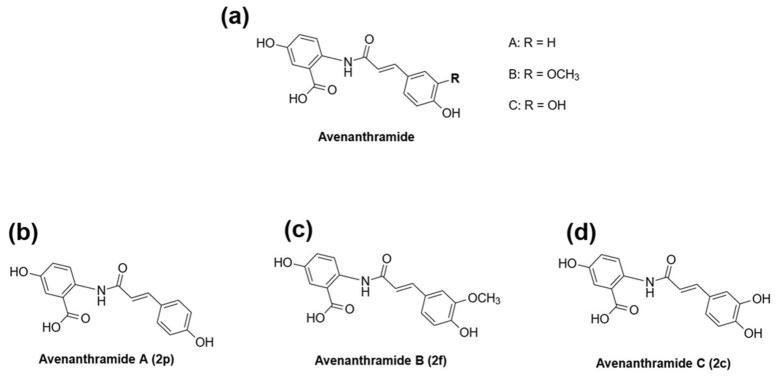
Chemical structures of the three major avenanthramides in oats: (**a**) general avenanthramide scaffold with variable R group substitution; (**b**) Avenanthramide A (2p, R=H); (**c**) Avenanthramide B (2f, R=OCH_3_); (**d**) Avenanthramide C (2c, R=OH).

The biosynthesis of these compounds begins with the phenylpropanoid pathway, a critical metabolic route for synthesizing phenolics involved in plant stress responses [117]. The first and rate-limiting step in this pathway is catalyzed by phenylalanine ammonia-lyase (PAL), which is the conversion of L-phenylalanine to trans-cinnamic acid [118,119,120]. Trans-cinnamic acid is subsequently converted into the key hydroxycinnamic acid precursors: *p*-coumaric acid, ferulic acid, and caffeic acid. These, in turn, form the core structures of the three major avenanthramides: *p*-coumaric (2p), caffeic (2c), or ferulic (2f) acid [49] (Figure 3).

In a study of three cultivars, ‘Dane’ exhibited a 125% increase in avenanthramide content, while ‘Vista’ showed a modest 29% increase, and ‘Gem’ displayed no significant change. Beyond total content, the profile also shifts; isoforms 2p and 2f typically increase significantly, whereas 2c remains stable [49,65]. A recent study by Živković et al. [52] examined common (‘Noni’) and naked (‘Kamil’) oat sprouts, showing 3.1- and 3.2-fold increases after 96 h at 20 °C, respectively. Interestingly, the 2c concentration exhibited a peak before declining after 48 h. The authors noted that differences in seed coat structure, affecting water and oxygen uptake, may potentially explain why the ‘Noni’ variety reached its maximum avenanthramide content sooner. Recent research reinforces the genotypic influence on avenanthramide accumulation. Jágr et al. [68] identified ‘CDC Boyer’, ‘Diadem’, and ‘Rozmar’ as optimal genotypes with the ‘Pennlo’ variety showing 293-fold increase to 1764 µg g^−1^ at 20 °C for 196 h, while ‘Shadow’ exhibited unique accumulation kinetics. The potential increase is profound under optimal conditions. Ding et al. [20] reported significant gains, with levels of avenanthramides 2c, 2p, and 2f peaking at 42.7, 25.9 and 18.6 times their original concentrations after 48 h of germination. Comparably, Huang et al. [67] recorded dynamic changes, with the three major avenanthramides reaching maxima of 4.8-, 3.3- and 2.5-fold increases after 3 days. This accumulation is attributed to the activation of hydroxycinnamoyl-CoA:hydroxyaminobenzoate N-hydroxycinnamoyltransferase (HHT), an enzyme that utilizes free hydroxycinnamic acid precursors, such as *p*-coumaric acid, to biosynthesize avenanthramides during sprouting [49,65].

Compared to non-sprouted oats (2–82 µg g^−1^), sprouted oats can achieve concentrations up to 293-fold higher depending on cultivar and germination conditions. Thus, sprouted oats offer a potent source of these unique oat bioactive compounds compared to their non-sprouted counterparts.

##### Synthesis and Implications for Avenanthramides

Sprouting can be employed to significantly enhance the levels of these oat-specific nutraceuticals, with optimal accumulation occurring at germination durations of 72 to 196 h and temperatures of 18 to 24 °C. The magnitude of this enhancement is strongly cultivar-dependent, with select varieties such as ‘Pennlo’ exhibiting 293-fold increases under optimized conditions [68]. The functional relevance of this enrichment is supported by emerging evidence of avenanthramide bioactivity in animal models. Zhang et al. [121] noted that high dose avenanthramides (100 to 300 mg kg^−1^ day^−1^) reduced weight gain and improved metabolic health in obese mice, highlighting the therapeutic potential of these compounds.

To maximize this benefit, processors must pair high-response oat cultivars with optimized germination conditions that promote HHT activity and precursor availability. This targeted approach can transform sprouted oats into a concentrated source of these health-promoting compounds. A key priority for future work is establishing cultivar-specific avenanthramide responses to enable predictable outcome-driven processing. Clinical validation of avenanthramide bioavailability and anti-inflammatory efficacy is addressed in Section 7.

### 5.7. Integrative Analysis of Germination Trade-Offs

The data presented across Section 5.1, Section 5.2, Section 5.3, Section 5.4, Section 5.5 and Section 5.6 reveal that no single germination condition simultaneously maximizes all nutritional outcomes. Shorter and cooler conditions (24 to 48 h, 16 to 18 °C) minimize β-glucan loss (≤20% degradation) and moderate phytate reduction (12 to 30%) but yield moderate increases in phenolics, GABA, and avenanthramides. Longer, warmer conditions (120 to 216 h, 20 to 24 °C) maximize bioactive accumulation (e.g., 31-fold GABA increase, 293-fold avenanthramide increase) and achieve near complete phytate reduction (80 to 85%) but result in substantial β-glucan degradation (up to 80%) and potential oxidative rancidity risks.

A balanced condition of 72 to 96 h at 18 to 20 °C represents a compromise for many applications: it achieves 2.5–3.2-fold increases in phenolics and GABA, 3- to 10-fold increases in avenanthramides, 40 to 60% phytate reduction, and significant improvements in protein digestibility, though approximately 50% β-glucan loss remains a consideration for products with cholesterol-reduction claims. Cultivar selection further modulates this balance; high-response varieties such as ‘Pennlo’ can achieve high avenanthramide enrichment under optimized conditions of 20 °C for 196 h [68].

Optimization must therefore be outcome driven. For products prioritizing β-glucan retention (e.g., cholesterol-reducing claims) or mineral bioavailability, short to moderate germination is preferred. For food products targeting maximal antioxidant and other health benefits, longer, warmer conditions with high-response cultivars are justified. Subsequent studies should systematically map these trade-off surfaces across multiple cultivars to enable predictive, multi-objective optimization of sprouted oat ingredients.

## 6. Effects of Storage and Processing on the Safety and Quality

### 6.1. Microbiological Safety

While sprouting enhances the nutritional value of oats, the process introduces significant microbiological safety concerns that must be managed. The warm, moist conditions ideal for germination are equally conducive to the proliferation of pathogenic bacteria, with common contaminants including *Salmonella* and *E. coli* O157:H7 [122,123]. Contamination can originate from the seed itself, the germination medium, handling, and storage making the process prone to risk [22].

Consequently, intervention strategies are critical. These are broadly categorized as physical (e.g., refrigeration, irradiation), biological (e.g., antagonist microbes, bacteriophages), and chemical treatments. Chemical disinfection, particularly with chlorine (50 to 200 ppm), is a widely adopted post-harvest method due to its broad-spectrum efficacy and practicality, though other methods exist [22,122,123]. Reflecting this standard, many of the biochemical studies cited in this review employed sodium hypochlorite for surface sterilization of oat seeds prior to germination to establish a controlled baseline (e.g., Aparicio-García et al. [34], Huang et al. [67]). The importance of such controls is highlighted in contrast to studies like Wilhelmson et al. [58], which observed a substantial increase in microbial counts (including *Fusarium*, aerobic heterotrophic bacteria, *Pseudomonas* spp., lactic acid bacteria, enterobacteria, and aerobic spore-forming bacteria) during oat germination at elevated temperatures without reported surface sterilization, underscoring how protocol differences directly influence microbiological outcomes.

Therefore, any protocol designed to optimize the nutritional benefits of sprouted oats, as detailed in preceding sections, must incorporate validated microbial intervention steps. Achieving this balance between maximizing bioactive compound enrichment and ensuring microbial safety is a fundamental challenge and a prerequisite for the successful development of safe, high quality sprouted oat ingredients and products.

### 6.2. Shelf-Life

The nutritional enhancements achieved through sprouting are offset by significant shelf-life challenges, primarily driven by the increased susceptibility of sprouted oats to lipid oxidation and associated sensory deterioration.

Literature demonstrates a clear pattern of quality decline during storage. Heiniö et al. [124] found that germinated oats deteriorated within the first six months of storage, with free fatty acid accumulation and lipid oxidation. Sensory panels described the stored products as musty, earthy, bitter, and rancid. This emphasizes the direct link between chemical degradation (lipid breakdown and oxidation) and negative sensory outcomes. A more accelerated study by Kince et al. [125], storing germinated oat flakes at 55 °C and 55% relative humidity for 6 months, confirmed that elevated lipid content drives oxidation and bitter off flavors, though these remained at acceptable levels within the test period. Notably, microbiological spoilage was not the limiting factor in either study.

The central implication is that oxidative rancidity, not microbial growth, is the primary shelf-life limiter for sprouted oat products. Consequently, preserving the quality of sprouted oats requires strategies that directly address this instability. As noted by Kince et al. [125], specialized packaging (high-barrier materials like Fibrecote^®^) is necessary, yet even this only extends shelf-life to approximately 10 months at 23 °C. Thus, for the commercial success of sprouted oat products, optimized packaging, controlled atmosphere storage, and potentially the use of natural antioxidants must be integral components of the post-germination processing chain to protect the very nutritional gains the sprouting process aims to achieve. Future research should quantify shelf-life stability across cultivars and storage conditions and evaluate natural antioxidants for extending shelf-life without compromising sensory quality.

### 6.3. Processing

The high-moisture, enzymatically active state of freshly sprouted oats necessitates immediate processing to stabilize the product for storage and consumption. Thermal treatment, primarily kiln drying, is the standard industrial method to achieve this. The primary goals are to inactivate spoilage enzymes (particularly lipases and lipoxygenases), reduce moisture to prevent microbial growth and thereby extend shelf-life [17,126].

However, this essential stabilization step presents a potential nutritional compromise. The application of heat, while crucial for deactivating rancidity-causing enzymes, may also degrade other heat-labile bioactive compounds that were enhanced during sprouting, such as certain vitamins, enzymes, and phenolic compounds. The degree of this impact is a function of the thermal load (temperature, time, and moisture during drying). Accordingly, the development of sprouted oat products requires a careful balance. Processing conditions must be optimized to deliver the minimum thermal input necessary for microbial safety and lipid stability, while maximizing the retention of the sensitive nutritional compounds that provide the primary benefit of sprouted oats. Future research should focus on precisely mapping this balance, exploring milder or alternative drying technologies (e.g., vacuum drying) to preserve the labile nutritional gains of the sprouting process.

## 7. Sprouted Oat Seeds and Human Health

The documented analysis of bioactive compounds during oat sprouting establishes a strong biochemical rationale for investigating its health benefits. However, it is important to emphasize that the current evidence base is predominantly preclinical, with limited clinical trials available to date. Most supportive data are derived from animal and in vitro models, which provide mechanistic insights but cannot directly predict human outcomes.

The unique oat phenolics, avenanthramides, are a primary focus. In rodent models of colon carcinogenesis, avenanthramides from germinated oats demonstrated significant chemopreventive properties, reducing tumor incidence and modulating inflammatory and redox enzymes [127]. Separately, avenanthramide 2c was shown to suppress allergic inflammation by inhibiting mast cell degranulation and the pro-inflammatory NF-κB pathways [128]. These findings highlight a multi-target anti-inflammatory capacity.

Beyond specific compounds, whole sprouted oat extracts show enhanced systemic bioactivity. In vitro studies using human liver (HepG2) cells confirm that germinated oat extracts more effectively reduce reactive oxygen species and activate cellular antioxidant defense pathways (Nrf2) compared to non-sprouted oats [129]. Furthermore, sprouted oats exhibit superior in vitro antioxidant and anti-hyperglycemic activity, with improved stability and polyphenol release during simulated intestinal digestion, suggesting better bioavailability [130]. These findings provide valuable mechanistic indicators, but they do not substitute for human clinical data.

The collective evidence indicates that sprouted oats are a source of bioavailable bioactive compounds with demonstrated activity effects against oxidation, inflammation, and metabolic stress. However, this preclinical foundation, while promising, requires validation in human populations. This provides a strong rationale for future human clinical trials. Future studies should prioritize: (1) intervention trials using well-characterized sprouted oat products to validate these benefits for cardiometabolic health, glycemic control, and gut health in human populations; (2) dose–response studies to establish the minimum effective intake of sprouted oat bioactives (e.g., avenanthramides, GABA) required to achieve the effects observed in preclinical models; and (3) human intervention trials to confirm that the anti-inflammatory and antioxidant pathways identified in vitro and in animal models are relevant in human physiology.

### Outlook for Nutraceutical Ingredients from Oats

The growing interest in health-promoting products has led to the coexistence of several related yet distinct categories: functional foods, nutraceuticals, and dietary supplements. These categories are distinguished by three criteria: form (conventional food or pharmaceutical delivery), purpose (general wellness or targeted therapeutic benefit) and regulatory context (food regulation, supplement, or drug).

The term ‘nutraceutical, the combination of ‘nutrition’ and ‘pharmaceutical’, was coined by Dr. Stephen DeFelice in 1989 to describe products derived from food sources that are sold in medicinal formats with intended therapeutic benefits, occupying a middle ground between food and drug [131]. This concept is often confused with functional foods; they are defined as conventional foods consumed as part of a regular diet that provide enhanced physiological benefits. On the other hand, dietary supplements are concentrated sources of nutrients or other substances intended to supplement the diet [132].

As summarized in Table 3, the key distinctions often lie in their form, purpose, and regulatory context. Functional foods (probiotic yoghurt) are consumed as part of a normal diet. Nutraceuticals (ginseng capsules) are food-derived compounds delivered in pharmaceutical forms like pills for targeted health benefits, including disease prevention and management with potentially fewer side effects [133]. Dietary supplements (multivitamins) are dose-form products aimed at correcting nutritional deficiencies or supplementing intake.

The classification of nutraceuticals itself remains broad and can overlap with other categories, encompassing traditional and fortified products, phytochemicals, herbal extracts, as well as probiotics and prebiotics [133,134]. Despite the ambiguity, the nutraceutical field represents a significant and growing intersection of nutritional science and pharmaceutical research, driven by consumer demand for preventive health solutions.

**Table 3:** Distinction between functional foods, nutraceuticals, and dietary supplements.

	Definition	Regulation	Examples
Functional Foods	Functional foods are conventional foods consumed as part of a regular diet that offer in addition to nutrition, additional physiological benefit to the consumer, such as promoting health, preventing disease, or minimizing the effects of health concerns [132,135].	Not recognized as a distinct legal category; regulated through general food laws and health-claim legislation (EU: Regulation (EC) No 1924/2006; Australia/NZ: Standard 1.2.7), with classification dependent on claims.	Whole grains and fiber (digestive health), calcium-fortified milk (bone health), probiotic yoghurt drinks (enhance gut microbiota) [136].
Nutraceuticals	Nutraceuticals are products derived from food that are sold in medicinal forms, such as pills, capsules, or ampoules, and are intended to provide therapeutic effects or health benefits [131,133].	Not recognized as a distinct legal category; regulated through general food laws and health-claim regulations (EU: Regulation (EC) No 1924/2006 and Regulation (EU) 2015/2283 where applicable; Australia/NZ: Standard 1.2.7), with classification dependent on claims.	Omega-3 fatty acid (heart health), probiotics (gut health), ginseng (cognitive function) [137].
Dietary (Nutritional) Supplements	Dietary supplements are products taken in the form of tablets, capsules, powders, or liquid drinks. They are designed to supply essential nutrients that may be deficient in the human body [132,138].	Recognized as a distinct regulatory category, but requirements vary by jurisdiction. EU: Directive 2002/46/EC and EC No 1924/2006; Australia/NZ: FSANZ or TGA, depending on claims; US: DSHEA (1994).	Multivitamins, oil supplements, herbal supplements (maintain nutrient adequacy and general wellness) [132,138].

Note. Abbreviations: EU = European Union; EC = European Community; NZ = New Zealand; FSANZ = Food Standards Australia and New Zealand; TGA = Therapeutic Goods Administration; US: United States of America; DSHEA: Dietary Supplement Health and Education Act.

As illustrated in Table 4, oats contain a diverse matrix of bioactive compounds with significant health potential. To strategically enhance this potential for nutraceutical applications, bioprocessing techniques such as germination (sprouting) are employed. Sprouted oats undergo enzymatic changes that can increase the concentration and bioavailability of key compounds including avenanthramides and GABA. For instance, avenanthramides are unique to oats and known for their anti-inflammatory and antioxidant activities (Table 4), are often found in increased concentrations post-germination. Furthermore, the sprouting process specifically induces the synthesis of GABA, a non-protein amino acid with demonstrated anxiolytic, hypotensive, and neuroprotective effects [97,139].

Hence, sprouted oats are not merely a nutrient-dense food but a value-added food where bioprocessing strategically amplifies specific health-promoting attributes. This makes them particularly suitable for incorporation into targeted nutraceutical formulations, such as capsules for cholesterol management (leveraging β-glucan and phytosterols), powders for metabolic health (utilizing enhanced phenolics and protein), or functional products for stress reduction (capitalizing on elevated GABA). The transition from whole oat to sprouted oat ingredient exemplifies the innovative development pathway within the nutraceutical sector, moving from generic nutritional benefit to tailored, bioactive-driven health solutions.

## 8. Conclusions and Future Trends

Oats represent a widely cultivated cereal grain, valued not only as a dietary staple but also as a rich source of beneficial nutrients and bioactive compounds. Public and scientific interest in oat products has surged due to their demonstrated potential in reducing cholesterol absorption, anti-cancer effects, and supporting gastrointestinal health.

This review highlights that the traditional practice of germination serves as a biotechnological tool to strategically enhance the nutraceutical potential of oats. This process fundamentally alters the oat matrix, softening the kernel, degrading antinutrients, and inducing the accumulation of health-promoting compounds such as avenanthramides, GABA, and improved amino acid profiles, while enhancing mineral bioavailability. The evidence suggests that these biochemical shifts translate to measurable health benefits, including enhanced antioxidant, anti-inflammatory and cardio-metabolic protective effects. Importantly, the germination process can be optimized through controlled biotic and abiotic elicitors to tailor and amplify the production of specific bioactive compounds, enabling the active design of nutraceutical ingredients.

However, several critical limitations must be acknowledged. First, most of the evidence is derived from in vitro and animal studies; human clinical trials remain scarce. Second, the influence of key germination parameters—particularly light spectra and cultivar-specific responses—remains poorly characterized. Third, the trade-offs between nutritional enhancement (e.g., increased TPC, reduced β-glucan) and product stability (oxidative rancidity, sensory deterioration) have not been quantified.

To address these limitations, targeted future research is essential. While preclinical evidence is promising, clinical trials are urgently required to validate the specific health impacts of sprouted oats in human populations. Future research should address key mechanistic and applied gaps: (1) cultivar-specific optimization is required to establish response curves for key bioactive compounds across commercially relevant oat varieties; (2) balancing nutritional gains with stability by defining processing windows that maximize target compounds while preserving shelf-life and sensory quality, integration of post-sprouting stabilization where necessary; (3) human validation through intervention trials using well-characterized sprouted oat products is needed to confirm cardiometabolic, glycemic, and gastrointestinal health benefits; and (4) quantifying the bioaccessibility and storage stability of key bioactive compounds in final food and nutraceutical formulation using standardized in vitro and accelerated shelf-life protocols.

Sprouted oats are positioned to transition from a traditional product into a versatile, value-added ingredient for the functional food and nutraceutical sectors. While further clinical and mechanistic validation is needed, the integration of traditional knowledge and modern bioprocessing positions germinated oats as a promising platform for developing targeted, evidence-based nutritional solutions aimed at chronic disease prevention and health promotion.

## Figures and Tables

**Table 1:** Effect of germination on nutritional properties under various sprouting conditions in oats.

Cultivar/Genotype	Steeping Conditions	Temperature (°C)	Duration (h)	Key Findings	References
Baton, NO3-6, Pennuda, MF9018-5901, MF9018-11801, 87Ab5932, 90Ab1500, etc.	16 °C for 16 h, with a 0.5 h air rest at 12 h	16	144	Starch: average decrease of 4.5%; β-Glucan: 4.84% → 0.3% (−93%); FFA: increased significantly; Nitrogen & lipid content showed modest changes.	[55]
Shaanxi	16 °C for 24 h (dark)	16	0, 24, 48, 72, 96, 124, & 144	Key increases (at 144 h): Protein: 18.98 → 22.02% (+16%); FAA: 0.03 → 0.37% (+~10-fold); Thr: 0.52 → 0.70 g 100 g^−1^ (+35%); Iso: 0.38 → 0.78 g 100 g^−1^ (+2-fold); Lys: 0.9 → 1.22 g 100 g^−1^ (+35%); Free sugars: 5.23 → 28.11% (+5.2-fold); TPC: 0.20 → 0.91% (+>4-fold) (120 h). Key decreases: Starch: 59.80% → 20.87% (−65%); Phytate: 0.35 to 0.11% (−68%).	[56]
Chimene	RT for 4 h	21	120	Key increases: Free phenols: 32.10 → 76.62 m g 100 g^−1^ GAE (+2.4-fold); Bound phenols: 60.45 → 124.36 mg 100 g^−1^ GAE (+2-fold); TPC: 23.08 → 386.71 mg 100 g^−1^ (+16.7-fold); Total AVN (2c, 2p, and 2f): +39.2%; MUFA & SFA: +20%; Oleic acid: +15%; Stearic acid: N.D → 2.04%. Key decreases: TDF: 12.63 → 8.81 g 100 g^−1^ (−30%); β-glucan: 4.46 → 1.55 g 100 g^−1^ (−75%); Linoleic acid: −2.6%; Phytic acid: 0.94 → 0.83 g 100 g^−1^ (−12%).	[50]
Hard red winter oats	15 °C for 48 h	15	0, 48, 96, 144, 192, 240, 288 & 336	Key findings (at 336 h): α-amylase activity: 1.0 → 61.2 U g^−1^ (+61-fold); Free sugars: 7.5 → 45.9 mg g^−1^ (+~6-fold); Damaged starch content: 6.1 → 9.6% Farrand (+~1.5-fold); starch granules visibly disintegrated.	[57]
Unknown	23–24 °C for 4 h	24 ± 2	24, 48, 72, & 96	Key increases (at 96 h): Sugars: Maltose: 31.39 → 3579.27 µg 100 mg^−1^ (+114-fold); Glucose: 258.02 → 5608.38 µg 100 mg^−1^ (+21.7-fold). Bioactive compounds: GABA: 9.47 → 297.69 µg 100 mg^−1^ (+31.4-fold); Total AVN (2c, 2p, and 2f): 17.07 → 312.98 µg g^−1^ (+18.3-fold); Glu: 93.56 → 701.41 µg 100 mg^−1^ (+7.5-fold). Organic acids: α-ketoglutaric acid: 7.06 → 34.07 µg 100 mg^−1^ (+4.8-fold); Succinic acid: 16.08 → 54.36 µg 100 mg^−1^ (+~3.4-fold). Antioxidants: TPC +3.2-fold; DPPH scavenging activity (at 24 h): +39%.	[20]
Meeri (Dehulled)	20 °C for 4 h	18	96	Key increases: Protease (+3-fold); α-amylase (+42-fold). GABA: 16.71 → 54.92 mg 100 g^−1^ (+~3.2-fold); Antioxidant activity: 585.26 → 1744.27 mg TE 100 g^−1^ (+~3-fold); FPC: 202.92 → 507.39 mg GAE 100 g^−1^ (+2.5-fold). Key decreases: β-glucan: 3.48 → 2.10 g 100 g^−1^ (~40%). Notable shifts: Gly 8.25 → 4.02 g 100 g^−1^ (−51%); Ala: 5.84 → 4.43 g 100 g^−1^ (−24%); Met: 0.42 → 0.81 g 100 g^−1^ (+1.9-fold); Na: 6.27 → 143.73 mg kg^−1^ (+22.9-fold).	[18]
Unknown	Varying wet and dry phases 3 h duration (7 cycles)	10, 12, 15, 18, & 20	48, 62, 96, 130, & 144	Key increases: IDF (+1.4-fold at 20 °C/144 h). Minerals: Ca: 20.8 → 32.4 mg 100 g^−1^ at 12 °C/96 h (+1.60-fold); Cu: 0.2 → 1.5 mg 100 g^−1^ at 15 °C/96 h (+6.5-fold). Key decreases: Phytate: 0.683 → 0.467% at 10 °C/144 h (−28%); SDF: 3.4 → 1.2% at 18 °C/96 h (−65%); β-glucan: 4.2 → 0.2% at 10 to 18 °C/96 to 144 h (−95%).	[35]
Meeri (Dehulled) Barra (Hulled)	20 °C for 4 h	12, 14, 16, 18, & 20	24, 60, 96, 156, & 216	Key increases: Protein: Barra: 8.42 → 12.69 g 100 g^−1^ at 16 °C/216 h (+1.5-fold). FPC: Meeri: 221.87 → 589.41 mg GAE 100 g^−1^ at 18 °C/156 h (+~2.7-fold). Antioxidant capacity: Barra: 574.58 → 1563.94 mg TE 100 g^−1^ at 16 °C/216 h (+2.7-fold); Meeri: 537.79 → 2525.07 mg TE 100 g^−1^ at 18 °C/156 h (+2.7-fold). Enzymes: α-amylase: Barra: 0.16 → 30.14 U g^−1^ at 14 °C/156 h (+188-fold); Protease: Barra: 0.28 → 10.88 U g^−1^ at 16 °C/216 h (+16-fold); Meeri: 0.27 → 5.07 U g^−1^ at 18 °C/156 h (+18-fold); Lipase: Barra: 3.26 → 7.34 U g^−1^ at 20 °C/96 h (+2.2-fold). Key decreases: β-glucan: Barra: 2.03 → 0.72 g 100 g^−1^ at 18 °C/156 h (−~65%); Meeri: 3.45 → 1.67 g 100 g^−1^ at 16 °C/216 h (−~52%). Lipase: Meeri: 3.55 → 2.76 U g^−1^ at 20 °C/96 h (−22%). Other notes: Large protein bands (>40 kDa) degraded with longer, warmer germination.	[34]
Veli (Dehulled) Lisbeth (Hull-less)	RT for 8 h followed by air rest for 16 h (repeated twice).	5, 15, 25	288 h at 5 °C, 192 h at 15 °C, 72 h at 25 °C	Key increases (at 15 °C/192 h): Enzymes: α-amylase: Veli: 1 → 139 U g^−1^ (+139-fold), β-glucanase: Lisbeth: 29 → 586 U g^−1^ (+~20-fold). Key decreases: β-glucan (Content): At 15 °C/192 h: 5.2 → 0.2% (−96%), Veli: 3.2 → 0.2% (−~94%). β-glucan (molecular weight): At 5 °C/288 h, Lisbeth M_w_: 2.5 × 10^6^ → 0.9 × 10^6^ (−64%); M_n_: 0.31 × 10^6^ → 0.05 × 10^6^ (−~84%). Other notes: Elevated temperatures increased microbial counts (*Fusarium*, aerobic heterotrophic bacteria, *Pseudomonas* spp., lactic acid bacteria, enterobacteria, & aerobic spore-forming bacteria).	[58]
Terra	RT for 24 h	23 with light, 20 and 25 in the dark.	24, 48, 72, 96, 120, 144, 168, & 192	Key increases: Nitrogen content: 2.84 → 3.19% at 25 °C/192 h (+1.1-fold); Non-protein nitrogen: 10 → 26% at 23 °C/192 h (+2.6-fold); Lys: 4.4 → 5.3 g per 16 g N at 23 °C/192 h (+1.2-fold). Key decreases: Dry matter: −20% (at 20–25 °C/192 h). Total Nitrogen (amount): 73.2 → 62.4 mg at 20 °C/192 h (−15%). Storage Proteins (at 23 °C/192 h): Globulin: 41 → 25% (−39%); Prolamin: 14 → 7% (−50%). EAAs (at 23 °C/192 h): Phe + Tyr: 9.2 → 7.3 g per 16 g N (−20%); Met + Cys: 4.2 → 2.6 g per 16 g N (−38%).	[59]
Unknown	18 °C for 12 h	20	48	Key increases: Amino acids: Total EAA: 130.68 → 602.68 mg g^−1^ protein (+4.6-fold); Total Non-EAA: 258.91 → 978.74 mg g^−1^ protein (+~3.8-fold); TAA content: 389.60 → 1581.41 mg g^−1^ protein (+~4-fold). Total soluble protein: 28.10 mg g^−1^. Bioactive compounds: GABA = +20%; TPC: 13.52 mg g^−1^; Total Flavonoid: 7.15 mg g^−1^. Antioxidants capacity: ABTS scavenging: 86.35%.	[33]
Unknown	Varying wet stage at 13 °C (6 h, 4 h, 3 h) and an air rest stage at 18 °C (10 h, 7 h, 1 h)	6 h at three stages at 13, 15 & 17	120	Key increases: FAAs: Thr: +~31-fold; Phe: +~22-fold); Pro: +664-fold; Tyr: +42-fold. Protein fractions: Albumin: Phe: 9.73 → 11.68 mg g^−1^ (+1.2-fold). Proteolytic activity: 2.4 → 16.3 mg h^−1^ g^−1^ (+6.8-fold). Key decreases: Total AAs: Tyr: 2.87 → 1.25 mg g^−1^ (−56%). Protein fractions: Albumin: Lys: −15%; His: −15%; Arg: −21%. Prolamin: Phe: −~15%; Glu: −16%; Total AAs: −12%. Glutelin: Lys: −50%; His: −16%; Ser: −16%; Glu: −15%; Total AAs: −12%. Other notes: SDS-PAGE analysis confirmed proteolysis with major protein bands and the appearance of new lower molecular weight peptides.	[60]
Gehl	4.5 h wet steeping, 19 h air rest, and 4 h steeping, at 20 °C	10, 14, 20, 25, & 30	24, 48, & 72	At 72 h of germination: Key increases (at 20–25 °C): α-amylase activity; Reducing sugar content; Ascorbic acid content. Key decreases: β-glucan content (reduction of 3.9% at 20 °C); slight decrease at 10–14 °C) Notes: At 30 °C, changes were minimal with no notable synthesis of ascorbic acid.	[19]
Unknown (Hulled)	20 °C for 7 h	20	24, 48, 60, 72, 84, & 96	Key increases (at 96 h): FAA: 1994 → 6445 mg kg^−1^ at 96 h (+3.2-fold). Sugars: Glucose: 1.7 → 9.6 g kg^−1^ (+5.6-fold); Maltose: 4.8 → 7.4 g kg^−1^ (+1.5-fold). Key trends: Fructose decreased initially (4.5 → 1.2 g kg^−1^ at 48 h) before recovering to 3.4 g kg^−1^ by 96 h. Effect on processing contaminants (upon heating): Acrylamide concentration was 30% higher in sprouted vs. native oats after 5 min of heating, but 36% lower after 10 and 15 min of heating.	[61]
Baiyan II	20 °C, with aeration for 1 h every 4 h, 3 samples were carried out at 8, 16, and 24 h	16	12, 24, 36, & 48	At 48 h of germination: Key increases: Phenolic content: Free: 0.43 → 1.74 mg GAE g^−1^ (+4-fold); Total: 1.65 → 2.62 mg GAE g^−1^ (+1.6-fold). Specific compounds: *p*-coumaric acid (+~1.8-fold); Ferulic acid (+~1.7-fold); AVN (2p): (+1.9-fold). Antioxidant capacity (FRAP): 3.37→ 18.35 µmol Fe g^−1^ (+5.4-fold); Total: 14.67 → 26.2 µmol Fe g^−1^ (+1.8-fold). Key decreases: Phenolic content: Bound: 1.23 → 0.87 mg GAE g^−1^ (−29%); Gallic acid: 113.3 → 97.8 µg g^−1^ (−~14%). Antioxidant capacity: DPPH: Bound: (−52%); Total: (−46%); FRAP (Bound): −~27% at 36 h.	[49]
Bayou I Baiyan II	20 °C for different durations 2, 4, and 7 h	20	7, 13, 25, 37, 48, 60, & 72	Key increases (at 72 h): GABA: Baiyan II: 0.54 → 14.84 mg 100 g^−1^ (+27-fold); Bayou I: 1.41→ 14.84 mg 100 g^−1^ at 72 h (+14-fold). Both cultivars: TAA levels increased significantly during steeping and germination.	[62]
Unknown	1 h	19–23	72	Key increases: Total Lipid content: 1.76 → 3.85% (+2.2-fold); Linoleic acid (C18:2): 2.08 → 2.75% (+1.3-fold); Lignoceric acid (C24:0): 0.31 → 0.53% (+1.7-fold) Key improvement: Omega-6/Omega-3 ratio: 24 → 19 (−20%).	[63]
Lizete	22 °C for 24 h	35	12, 24, 36, & 48	Key increases: TPC: Significant increase observed after >36 h. Antioxidant capacity: Significant increase in both DPPH and ABTS radical scavenging capacities >12 h. Note: No significant increase in TPC was detected within the first 24 h.	[64]
Unknown	RT for 8 h, drained for 2 h and soaked for 1 h	11, 15, 37, & 40	30–40 120–168	Key finding: Phytate content is effectively reduced through controlled germination, with the extent of reduction dependent on time-temperature. Key reductions: 80–85% reduction: Achieved via malting for 5–7 days, including a 4 h steep at pH 5 and 37 °C. 64% reduction: Achieved via two-stage process (40 h at 15 °C + 20 h at 55 °C). ~16% reduction: Achieved via a single-stage incubation (30–40 h at 15 °C) Optimal protocol: Near complete phytate removal was achieved by germinating at 11 °C for 5 days, followed by incubation at 37– 40 °C for 17 h.	[36]
Vista, Gem & Dane	16 or 20 °C, 10, 12, and 14 h for Vista, Gem, and Dane, respectively	16 & 20	72 & 120	Key increases (at 16 °C/120 h): Total AVN: Dane: 63 → 142 nmol g^−1^ (+~2.3-fold); Vista: 68 → 88 nmol g^−1^ (+1.3-fold). HHT activity: +62%. Key decreases (at 16 °C/120 h): Hydroxycinnamic acid (Caffeic acid + *p*-coumaric acid): Decreased to an almost undetectable levels in all three cultivars. Phenoloxidase activity (at 16 °C/96 h): Vista −18%; Gem −23%; Dane 8%.	[65]
Choyang	25 °C for 8 h	25	60	Key increases: Color properties (b*): 10.8 → 22.4 (+2-fold); Gelatinization enthalpy (Δ*H*): 5.4 → 6.5 J g^−1^ (+1.2-fold). Key decreases: Amylose content: 17.2 → 14.3% (−17%). Color properties: L*: 87 → 77.3 (−11%); a*: 0.4 → −2.0 (−600%). Gelatinization range (*T_c_–T_o_*): 13.1 → 11.2 °C (−14%). Pasting properties: Pasting temperature: 92.3 → 67.1 °C (−27%); Peak viscosity: 479 → 46 cP (−90%); Breakdown: 181 → 36 cP (−80%); Setback: 919 → 9 cP (−99%); Final viscosity: 1217 → 20 cP (−98%). Swelling factor & amylose leaching: Decreased significantly with time.	[29]
Unknown (Hull-less)	RT for 8 h	16–18	40–50	Key increases: Energy value: 1532.7 → 1674.3 kJ 100 g^−1^ (+1.1-fold); Carbohydrates: 54.8 → 65.2 g 100 g^−1^ (+1.2-fold). Antioxidant capacity: DPPH: 0.4 → 4.6% 100 g^−1^ (+11.5-fold); FRAP: 2.3→ 6.7% 100 g^−1^ (+2.9-fold). Key decreases: β-glucan: 4.0 → 2.2% 100 g^−1^ (−43.7%). Note: Sensory was favorable due to the brittle structure.	[66]
Kamil (*A. nuda*) Noni (*A. sativa*)	RT for 8 h with h intervals of 15 min aeration	20	48, 72, 96 and 120	Key increases (in shoots, 72–120 h): Phenolics & Antioxidants: TPC (Noni): 9.3 → 22.31 mg FAE g^−1^ (+2.4-fold); DPPH activity (Noni): 3.76 → 10.52 TE g^−1^ (+2.8-fold). Total AVN: Noni: (+3.1-fold); Kamil: (+3.2-fold). Bound flavonoids (at 120 h): Isoschaftoside: Kamil: 8.44 → 248.81 µg g^−1^ (+29-fold); Noni: 5.95 → 349.79 µg g^−1^ (+~59-fold). Vitexin: Kamil: 1.76 → 11.09 µg g^−1^ (+6.3-fold); Noni: 1.08 → 10.9 µg g^−1^ (+10-fold). Note: Shoots biomass increased as kernel reserve mobilized. Phenolic and antioxidant capacity increased in both cultivars, with new compounds synthesized.	[52]
Unknown	20 °C for 20 h (aeration every 10 min)	25	72 h at 25 °C (RH ≥ 99%), then incubated for another 96 h at 30 °C (RH 70–80%),	Key findings: AVN diversity and synthesis: 28 distinct AVNs identified in seedlings, including a novel compound (6f). Total AVN content: +25-fold. The major AVN (2p, 2c, and 2f) comprised <20% of the total profile. Notably, the differences in composition between oat bran and oat seedlings indicate that the avenanthramide profile can vary between oat tissues.	[51]
Unknown	RT for 7 h	23	72, 120, 168 and 216	Key increases: TPC (at 216 h): 4.22 mg GAE g^−1^. Antioxidant capacity (at 216 h): DPPH: 7259.41 µg Trolox g^−1^ (+10-fold); ABTS scavenging: 6601.42 µg Trolox g^−1^ (+6-fold). AVN (at 72 h): 2c: 177.45 → 846.05 µg g^−1^ (+4.8-fold); 2p: 191.08 → 625.13 µg g^−1^ (+3.3-fold); 2f: 228.12→ 559.21 µg g^−1^ (+2.5-fold). Key decreases (72–216 h): Soluble protein: 16.03 → 7.99 mg g^−1^ (−50%); β-glucan: 2.5 → 0.24% (−90%); Cellulose: 8.93 → 4.16% (−53%). Note: Oat extracts (20–400 μg mL^−1^) maintained cell viability (>80%, RAW264.7). Germination time altered the activated antioxidant pathway (72 h: AMPK/HO-1; 216 h: PI3K/NQO-1).	[67]
Choyang	RT for 8 h in the dark	25	60	Key findings: Unlike other grains, no significant reduction in amylose content or molecular weight after germination. Key structural & functional changes: Starch granules: Morphology (oval/polyhedral) unchanged until >60 h of germination. Molecular structure: Amylopectin branch chains lengthened; crystallinity decreased. Pasting properties: Peak viscosity (at 24 h): 914 → 1041 cP (+1.1-fold); Setback (at 60 h): 1229 → 1094 cP (−10%). Slight decreases in swelling factor & amylose leaching.	[53]
AC Preakness (husked), Atego (husked), CDC Boyer (husked), Diadem (husked), etc.	20 °C for 24 h	20	24, 48, 72, 96, 120, 196	Key increase/findings: Total AVN: Pennlo: 6 → 1764 µg g^−1^ at 196 h (+293-fold). Most genotypes reached maximum between 48–72 h. Optimal varieties: CDC Boyer, Diadem and Rozmar were most suitable genotypes for maximizing AVN levels. Shadow: Showed unique kinetics: initial decreased (24–48 h), increase (72 h) with a partial decline (120 h), peaking at 192 h. AVN profile: Long-chain AVNs (2cd, 2fd, 2pd) were relatively abundant; Novel compounds (AVN 1a, 2a and 2ad) identified, Note: AVN accumulation is highly genotype dependent.	[68]

Note. Abbreviations: FFA = Free fatty acids; FPC = Free phenolic content; AVN = Avenanthramide; GABA = γ-Aminobutyric acid; TDF = Total dietary fiber; IDF = Insoluble dietary fiber, SDF = Soluble dietary fiber; FAA = Free amino acid; TAA = Total amino acid; EAA = Essential amino acid; AAs = Amino acids; M_w_ = Weight average molecular weight; M_n_ = Number average molecular weight. L*, a*, and b* are standard CIELAB color space coordinates.

**Table 2:** Summary of nutritional composition of sprouted oats (approximate ranges per 100 g).

Nutrient/Compound	Units	Non-Sprouted Oats	Sprouted Oats	References
**Macronutrients**				
Starch	g 100 g^−1^	51–65	20–60	[55,56,57]
Total protein	g 100 g^−1^	15–20	18–22	[18,34,56]
Total lipids	g 100 g^−1^	3–8	3–8 (variable)	[18,63]
Total dietary fiber	g 100 g^−1^	~12–14	8.8–14 (variable)	[35,50]
Β-glucan	g 100 g^−1^	3–8	0.2–3.5	[18,34,35,55,58]
**Bioactive**				
Total phenolic content	mg GAE 100 g^−1^	~200–222	~507–589	[18,34,35]
Antioxidant capacity	mg TE 100 g^−1^	~537–585	1563–2525	[18,34]
GABA	mg 100 g^−1^	~0.54–16.71	~55–300	[18,20,62]
Avenanthramides (Total)	mg 100 g^−1^	0.2–8.2	8.8–176.4	[67]
**Amino acids**				
Total essential amino acids	g 100 g^−1^	~18	~22	[49]
**Minerals**				
Calcium	mg 100 g^−1^	20.8–51.3	32.4–68.3	[18,35]
Magnesium	mg 100 g^−1^	114–158.7	130–191.3	[18,35]
Copper	mg 100 g^−1^	0.2	1.5	[35]
Phytate/Phytic acid	g 100 g^−1^	0.35–0.94	0.11–0.83	[35,36,50,56]

Note. Abbreviations: GAE = Gallic acid equivalents; TE = Trolox equivalents. Ranges compiled from Table 1.

**Table 4:** Overview of bioactive compounds in oats and their potential health benefits.

Active Ingredient	Potential Health Benefits	References
Lignans	- Anti-inflammatory, antioxidant and antitumor properties. - Reduction in cardiovascular diseases.	Rodríguez-García et al. [140], Peterson et al. [141]
Proteins and peptides	- Antioxidants, antihypertensive, antifatigue, antidiabetic, hypercholesterolemic, anti-hypoxic, antithrombosis and immunomodulatory activities.	Rafique et al. [142]
Phytosterols	- Reduction in serum low density lipid cholesterol.	Theuwissen and Mensink [143]
Tocopherols and tocotrienols	- Inhibit the proliferation of certain cancer cells. - Antioxidant properties. - Prevention of cardiovascular diseases. - Anti-cancer effects.	Martínez-Villaluenga and Peñas [144] Shibata et al. [145], Ramanathan et al. [146] Abraham et al. [147], Constantinou et al. [148]
Avenanthramides	- Antioxidant, anti-inflammatory and anti-proliferative activities.	Soycan et al. [114], Martínez-Villaluenga and Peñas [144]
β-glucan	- Reduction in blood glucose and serum cholesterol. - Reduction in systolic and diastolic arterial blood pressure. - Hypocaloric interventions. - Anti-inflammatory and chemopreventative effects.	Martínez-Villaluenga and Peñas [144] Evans et al. [149] Tong et al. [150] Zhou et al. [151], Choromanska et al. [152]
GABA	- Hypnosis, anxiolysis, muscle relaxation, anti-convulsant activities, stimulant, and cognitive enhancement. - Reduce blood pressure, prevent alcohol-related diseases, and inhibit cancer cell proliferation.	Enna and McCarson [97], Boonstra et al. [139] Xu et al. [62]

## Data Availability

No new data were created or analyzed in this study.
